# Assessing endocrine and immune parameters in human immunodeficiency virus-infected patients before and after the immune reconstitution inflammatory syndrome

**DOI:** 10.20945/2359-3997000000010

**Published:** 2018-01-01

**Authors:** Liliana Rateni, Sergio Lupo, Liliana Racca, Jorge Palazzi, Sergio Ghersevich

**Affiliations:** 1 Universidad Nacional de Rosario Universidad Nacional de Rosario Facultad de Ciencias Médicas Rosario Santa Fe Argentina Facultad de Ciencias Médicas, Universidad Nacional de Rosario, Santa Fe, Rosario, Argentina; 2 Center for Assistance and Comprehensive Clinical Research Rosario Mendoza Argentina Center for Assistance and Comprehensive Clinical Research (CAICI), IICTlab, Mendoza, Rosario, Argentina; 3 Universidad Nacional de Rosario Universidad Nacional de Rosario Facultad de Ciencias Bioquímicas y Farmacéuticas Rosario Argentina Facultad de Ciencias Bioquímicas y Farmacéuticas, Universidad Nacional de Rosario, Rosario, Argentina

**Keywords:** HIV, interleukins, immune reconstitution syndrome, cortisol, dehydroepiandrosterone sulfate

## Abstract

**Objective:**

The present study compares immune and endocrine parameters between HIV-infected patients who underwent the Immune Reconstitution Inflammatory Syndrome (IRIS-P) during antiretroviral therapy (ART) and HIV-patients who did not undergo the syndrome (non-IRIS-P).

**Materials and methods:**

Blood samples were obtained from 31 HIV-infected patients (15 IRIS-P and 16 non-IRIS-P) before ART (BT) and 48 ± 2 weeks after treatment initiation (AT). Plasma Interleukin-6 (IL-6) and Interleukin-18 (IL-18) were determined by ELISA. Cortisol, dehydroepiandrosterone sulfate (DHEA-S) and thyroxin concentrations were measured using chemiluminescence immune methods.

**Results:**

Concentrations of IL-6 (7.9 ± 1.9 pg/mL) and IL-18 (951.5 ± 233.0 pg/mL) were significantly higher (p < 0.05) in IRIS-P than in non-IRIS-P (3.9 ± 1.0 pg/mL and 461.0 ± 84.4 pg/mL, respectively) BT. Mean T4 plasma level significantly decreased in both groups of patients after treatment (p < 0.05). In both groups cortisol levels were similar before and after ART (p > 0.05). Levels of DHEA-S in IRIS-P decreased AT (1080.5 ± 124.2 vs. 782.5 ± 123.8 ng/mL, p < 0.05) and they were significantly lower than in non-IRIS-P (782.5 ± 123.8 vs. 1203.7 ± 144.0 ng/mL, p < 0.05). IRIS-P showed higher values of IL-6 and IL-18 BT and lower levels of DHEA-S AT than in non-IRIS-P.

**Conclusion:**

These parameters could contribute to differentiate IRIS-P from non-IRIS-P. The significant decrease in DHEA-S levels in IRIS-P after ART might suggest a different adrenal response in these patients, which may reflect the severity of the disease.

## INTRODUCTION

The immune reaction to different diseases elicits an endocrine response which influences the course of the process (1). In inflammatory processes, proinflammatory cytokines (besides their immunological effects) are known to affect the function of crucial neuroendocrine mechanisms, which, in turn, can modulate the immune response (2,3). Such mechanisms include the actions of cytokines on the hypothalamus-pituitary-adrenal (HPA), -gonadal and -thyroid axis (2,4).

Viral infections, in general, are physiologically stressful, as shown by the concomitant activation of the HPA axis, and it has become clear that cytokine-HPA axis interactions are fundamental for immune regulation during these infections (3,5). Among the pro-inflammatory cytokines, Interleukin-18 (IL-18) was shown to have an important role in the immune response to intracellular pathogens in acute infections and it may participate in the regulation of the HPA axis (6). Another pro-inflammatory cytokine, interleukin-6 (IL-6), can activate the HPA axis, leading to the final production of steroid hormones by the adrenal gland (7).

Glucocorticoids have a critical role in maintaining the balance between the beneficial and detrimental effects of pro-inflammatory cytokines as part of the bidirectional communication between the immune system and the HPA axis (3,8). Regarding the adrenal steroids, glucocorticoids can promote Th2 cytokine acquisition profile, facilitating Th2 activities, whereas dehydroepiandrosterone (DHEA) is able to favor Th1 related cytokine production and interferes with Th2 associated cytokine synthesis (9,10).

Some human immunodeficiency virus (HIV)infected patients undergo a clinical deterioration during the antiretroviral therapy (ART), which occurs regardless of the increase of CD4^+^ T lymphocyte counts and the decrease of plasma HIV-1 viral loads. This clinical condition, known as immune reconstitution inflammatory syndrome (IRIS), reflects an exacerbated inflammatory response to opportunistic pathogens and/or tumor antigens in HIV-infected patients (11,12). This disorder occurs after the initiation of ART and is temporally related to an increase in the host CD4^+^ lymphocyte count (11,13). The mechanisms involved in IRIS are not fully understood but they appear to be associated with the restoration of the immune response against pre-existent pathogens related to sub-clinical infections (14). The HIV-infected patients that will undergo IRIS during their treatment could present a more marked unbalance in their immune endocrine regulation (1,15).

Based on the mentioned data, the aim of the study was to assess parameters of adrenal and thyroid responses and immune pro-inflammatory reaction in HIV-infected patients receiving highly active ART. The results obtained from patients who suffered from IRIS (IRIS-P) and the ones who did not undergo the syndrome (non-IRIS-P) during treatment were compared in order to evaluate potential differences of the studied parameters between both groups of patients.

## MATERIALS AND METHODS

### Patients and ethics

All patients signed a written consent to participate in the study, and the protocol was approved by the Ethical Committee of CAICI Institute (Center for Assistance and Comprehensive Clinical Research, Rosario, Argentina). Patients with endocrine pathologies and hormonal treatments were excluded from the study.

This was a case-control study including 31 HIVinfected patients: 16 patients with normal response to ART (non-IP; 48 ± 11 years old), and 15 patients who underwent IRIS during the treatment (IP; 52 ± 12 years old). Both groups did not differ in age and sex composition (p > 0.05).

The diagnosis of IRIS was based on the criteria proposed by French and cols. (16), in patients who were infected with HIV and underwent a rapid clinical deterioration shortly after starting ART, despite having effective viral suppression. This was associated with co-infections caused by a diverse array of pathogens, and by tumor development. The diagnosis of IRIS was made by exclusion, ruling out other possible causes of disease after starting ART.

### Blood sample collection

Ethylenediaminetetra-acetic acid (EDTA)-treated blood samples were obtained from patients at 8:00 a.m, before treatment initiation and 48 ± 2 weeks after ART initiation. Following plasma separation and addition of aprotinin (100 U/mL, Sigma-Aldrich Inc, USA), samples were preserved at -20°C until used in the assays.

### T lymphocyte subsets count

T lymphocyte subsets (CD4, CD8) in patients’ blood samples were quantified by standard flow cytometry techniques. Fluorochrome-labelled antibodies (anti-CD8-fluorescein isothiocyanate isomer, anti-CD3-phycoerythrin, and anti-CD4-PE-Cy5, Becton Dickinson, Heidelberg, Germany) that specifically bind to lymphocyte surface antigens were added to aliquots of blood samples. After incubation, a fixative solution (Becton Dickinson) was added and sample analysis was performed on a Becton Dickinson FACSCALIBUR flow cytometer (Four-Colors; Becton Dickinson, Heidelberg, Germany). The analysis provided absolute counts of CD4^+^, CD8^+^, CD3^+^ lymphocytes and the CD4^+^/CD8^+^ ratio.

The absolute lymphocyte count was generated by a SYSMEX 2000i hematology analyzer (dual platform method, Roche, Basel, Switzerland).

### Viral load quantification

Total RNA was extracted from the patients’ samples and analyzed by the Amplicor HIV-1 Monitor test (Roche, Branchburg, NJ, USA) following the manufacturer's instructions.

### Assays of IL-6 and IL-18

Both IL-6 and IL-18 plasma concentrations were assayed by Quantitative Elisa Kits from R & D Systems (Minneapolis, MN, USA), following the manufacturer's instructions. The sensitivities of the assays were 0.7 pg/mL and 12.5 pg/mL, respectively. The intraassay variation coefficients for IL-6 and IL-18 assays were 5.0 % and 8.0 %, respectively.

### Hormone measurements

Plasma concentrations of cortisol, DHEA-sulfate (DHEA-S), and thyroxin (T4), were determined using an Immulite 1000 Immunoassay System (Siemens, USA). The intra-assay variation coefficients were always lower than 5.0%.

### Statistical analysis

Results from patients with and without IRIS or before and after ART were compared using the Student t-test or the alternative nonparametric Mann-Whitney test when required. Pearson's correlation coefficient (r) was used to analyze relationships among paired data. The Receiver Operating Characteristics (ROC) curve analysis was used to compare IL-18 values between IRIS-P and non-IRIS-P before the treatment. Results were expressed as media ± standard error (SE). A p < 0.05 was considered statistically significant.

## RESULTS

The following disorders were associated with the IRIS that suffered the IRIS-P: *Herpes zoster* infection, tuberculosis, hepatitis B, toxoplasmosis, polyarthritis, and Kaposi's sarcoma. These disorders appeared 5.0 ± 0.6 months after ART initiation and usually in a sequential rather than a concurrent way. No patient had an active disease or opportunistic infection at the time of testing for this study, i.e., before the treatment and after 48 ± 2 weeks of ART initiation.

### Immune parameters

The results indicated that all patients achieved a significant increase in their CD4^+^ T cell counts after treatment. The values of CD4^+^ T lymphocytes increased significantly after ART both in IRIS-P (p < 0.01) and in those who did not suffer from the syndrome (p < 0.01), compared to pre-treatment values (Table 1). The CD4^+^/CD8^+^ ratio was also significantly higher 48 ± 2 weeks after treatment initiation both in IRIS-P (p < 0.05) and in non-IRIS-P (p < 0.01). There were no significant differences in CD4^+^ - CD8^+^ cell counts or CD4^+^/CD8^+^ ratio between IRIS-P and non-IRIS-P, neither before nor after the treatment (Table 1).

**Table 1:** Measured parameters in HIV-infected patients, before ART or after 48 ± 2 weeks of treatment initiation

	BT		AT	
	IRIS-P SRI	Non-IRIS-P +/-SE	IRIS-P	Non-IRIS-P +/-SE
CD4^+^ (cel/mL)	221.4 ± 40.2^a^	262.3 ± 43.7^b^	447.5 ± 67.8^a^	429.8 ± 41.7^b^
CD8^+^ (cel/mL)	760.4 ± 152	919.0 ± 100.7	715.7 ± 107.1	965 ± 134.1
CD4^+^/CD8^+^	0.46 ± 0.22^c^	0.40 ± 0.15^d^	0.59 ± 0.11^c^	0.51 ± 0.06^d^
VL (copy number/mL)	261332 ± 117474	159954 ± 35324	167 ± 37	50 ± 0.0

The table shows the mean results of the measured parameters in IRIS-P and in non-IRIS-P, before treatment initiation (BT) or after 48 ± 2 weeks (AT) of ART initiation. Results were expressed as media ± SE. CD4^+^: CD4^+^ T lymphocytes counts. CD8^+^: CD8^+^ T lymphocytes counts.

VL: viral load.

The same letters indicate mean values which are significantly different:

a p < 0.01;

b p < 0.01;

c p < 0.05;

d p < 0.01.

Before treatment, the mean values of viral load in IRIS-P (261333 ± 117474 copy number/mL) and in non-IRIS-P (159954 ± 35324 copy number/mL) were not significantly different (Table 1).

**Figure 1:**
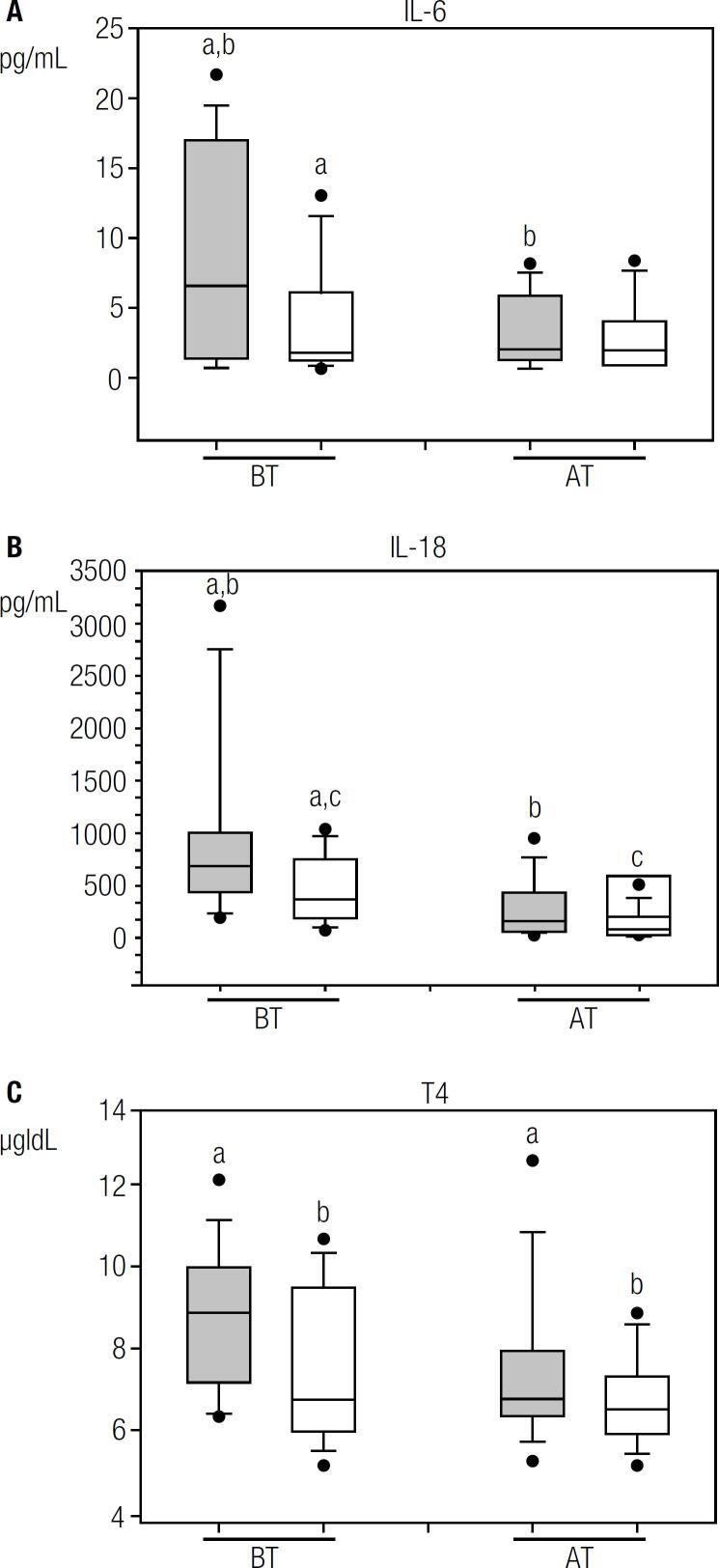
Box plots show plasma levels before (BT) or 48 ± 2 weeks after (AT) of ART initiation in IRIS-P (grey boxes) and non-IRIS-P (white boxes): A) interleukin 6 (IL-6, a: p < 0.05, b: p < 0.01); B) interleukin 18 (IL-18, a: p < 0.05, b: p < 0.05, c: p < 0.01); C) thyroxin (T4, a: p < 0.05, b: p < 0.05). The same letters indicate the groups that were compared statistically in the corresponding graph. Line inside box: median; limits of box: 75^th^ and 25^th^ percentiles.

Statistical analysis showed a significant difference in IL-6 mean plasma concentrations between IRIS-P and non-IRIS-P, before ART (p < 0.05, Table 2 and Figure 1A). In addition, in IRIS-P, the IL-6 values were significantly reduced after 48 ± 2 weeks after ART initiation (p < 0.01, Figure 1A) with respect to the values before the treatment. In the other patients, the decrease in IL-6 after ART did not reach statistical significance.

**Table 2:** Measured parameters in HIV-infected patients, before treatment initiation (BT) or after 48 ± 2 weeks (AT) of ART initiation

	BT		AT	
	IRIS-P SRI	Non-IRIS-P +/-SE	IRIS-P	Non-IRIS-P +/-SE
IL-6 (pg/mL)	7.9 ± 1.9^a,b^	3.9 ± 1.0^a^	3.2 ± 0.6^b^	2,8 ± 0.6
IL-18 (pg/mL)	951.5 ± 233.0^a,b^	461.0 ± 84.4^a,c^	270.4 ± 72.7^b^	30.7 ± 36.5^c^
Cortisol (µg/dL)	20.1 ± 1.5	20.3 ± 2.3	21.4 ± 2.5	21.0 ± 2.3
DHEA-S (µg/mL)	1080.5 ± 124.2^a^	1222.4 ± 1.7	782.5 ± 123.8^a,b^	1203.7 ± 144.0^b^
T4 (µg/dL)	8.9 ± 0.4^a^	7.8 ± 0.5^b^	7.2 ± 0.3^a^	6.9 ± 0.2^b^
DHEA/Cortisol	53.7 ± 6.5	66.2 ± 9.7	41.3 ± 7.9^a^	72.2 ± 11.9^a^

The table shows the mean results of the measured parameters in IRIS-P and in non-IRIS-P, before treatment initiation (BT) or after 48 ± 2 weeks (AT) of ART initiation. Results were expressed as media ± SE.

The same letters indicate mean values which were significantly different: IL-6: interleukin 6 (^a^ p < 0.05; ^b^ p < 0.01); IL-18: interleukin 18 (^a^ p < 0.05, ^b^ p < 0.05, ^c^ p < 0.01); DHEA-S: dehydroepiandrosterone sulfate (^a^ p < 0.05, ^b^ p < 0.05); DHEA-S/Cortisol ratio (^a^ p < 0.05); T4: thyroxin (^a^ p < 0.05, ^b^ p < 0.05).

Before ART, mean plasma IL-18 levels in IRIS-P were higher than those in patients who did not suffer from the syndrome (p < 0.05, Table 2, Figure 1B). Based on a ROC curve analysis, a IL-18 value of 695 pg/mL before ART would allow to differentiate IRIS-P from patients who did not undergo IRIS in the present study, with 80% of specificity and 57% of sensitivity (p = 0.08).

After 48 ± 2 weeks of ART initiation, the IL-18 values tended to be higher in IRIS-P than in the other patients (p = 0.09). Plasma concentrations of IL-18 before ART were significantly higher than those after treatment both in IRIS-P and non-IRIS-P (p < 0.05 and p < 0.01, respectively, Figure 1B).

### Endocrine measurements

Mean plasma concentrations of T4 in patients from each group are shown in Table 2, before and after the initiation of ART. Despite a significant decrease in the mean T4 plasma level after 48 ± 2 weeks of treatment in both groups of patients (p < 0.05, Figure 1C), the T4 concentrations always remained within the normal range. In addition, the mean values of T4 between the two groups, before or after ART, were not significantly different.

**Figure 2:**
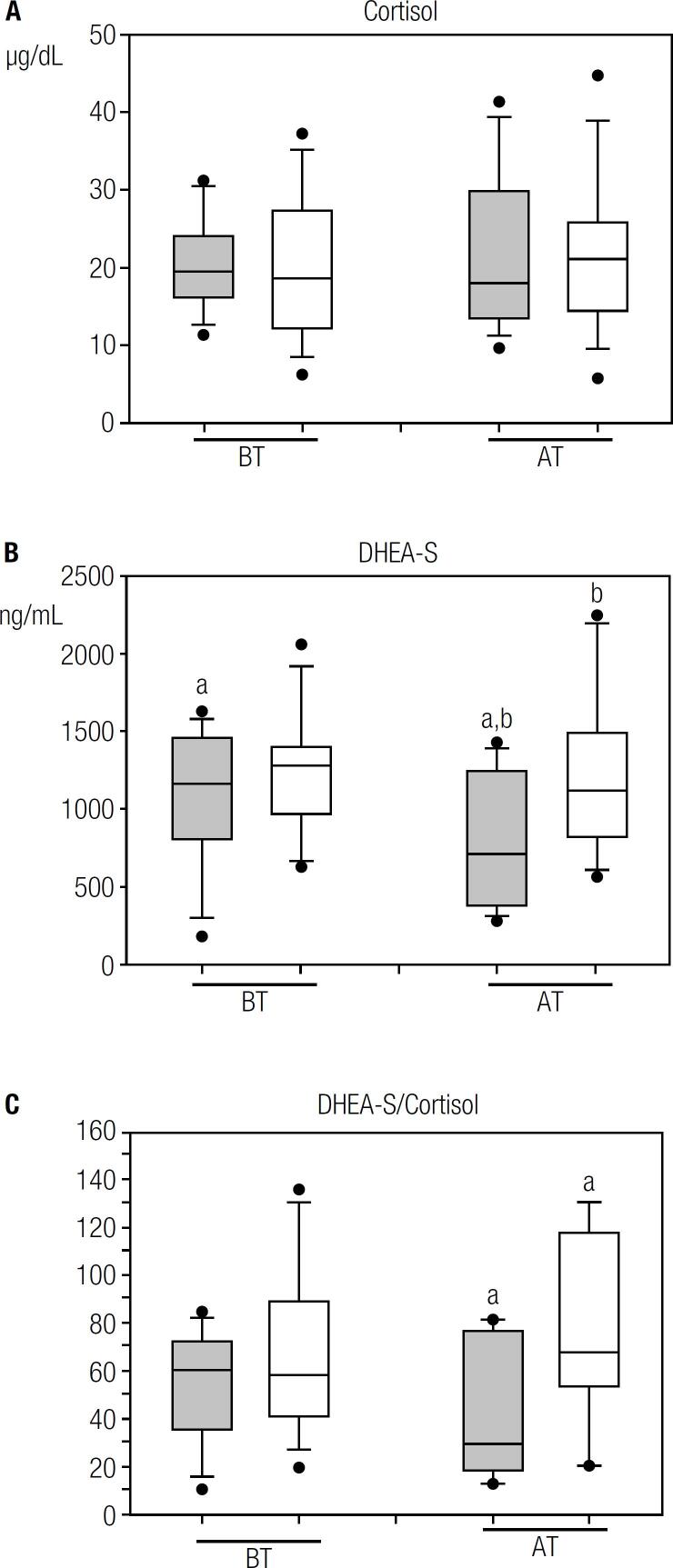
Box plots show plasma concentrations before (BT) or 48 ± 2 weeks after (AT) of ART initiation in IRIS-P (grey boxes) and non-IRIS-P (white boxes): A) cortisol; B) dehydroepiandrosterone sulfate (DHEA-S; a: p < 0.05, b: p < 0.05). C) Box plot indicates values of DHEA-S/Cortisol ratio before (BT) or after 48 ± 2 weeks (AT) of ART initiation in IRIS-P (grey boxes) and non-IRIS-P (white boxes, a: p < 0.05). The same letters indicate the groups that were compared statistically in the corresponding graph. Line inside box: median; limits of box: 75^th^ and 25^th^ percentiles.

Table 2 shows the mean plasma concentration values of cortisol found in IRIS-P and in the other patients. In both groups, cortisol levels were similar both before and after ART initiation (Figure 2A).

Before ART, a significant correlation (r = 0.59, p < 0.05) between the values of CD4^+^ lymphocytes count and cortisol plasma levels was observed in the patients who did not undergo IRIS. However, no association was found between CD4^+^ cell count and cortisol concentrations in IRIS-P before the treatment, nor between these parameters after ART initiation in both groups of patients.

The mean plasma DHEA-S concentrations significantly decreased after treatment in IRIS-P (p < 0.05; Table 2 and Figure 2B). Statistical analysis indicated a significant difference in mean plasma levels of DHEA-S between the IRIS-P and non-IRIS-P, after receiving ART (p < 0.05, Table 2 and Figure 2B). However, before ART, plasma levels of DHEA-S in IRIS-P were not different from the values in non-IRIS-P.

No significant correlation between the mean values of CD4^+^ cell count and DHEA-S plasma levels was found neither in IRIS-P nor in the other patients, neither before nor after ART initiation.

The statistical analysis did not reveal a significant difference in the values of DHEA-S/Cortisol ratio between the two groups of patients before ART (Table 2 and Figure 2C). However, after ART initiation, the values of DHEA-S/Cortisol ratio were significantly lower in IRIS-P (p < 0.05; Table 2 and Figure 2C) than the values observed in the other patients studied.

## DISCUSSION

It is known that treatment-induced immune improvement may increase the risk of an exacerbated immune response in some patients, worsening infections already present in the host, and leading to IRIS (12,17).

It has been reported that HIV-infected patients with a lower CD4^+^ cell count before ART initiation are at a higher risk of undergoing IRIS (18,19). However, in the present study there were no significant differences in CD4^+^ - CD8^+^ cell counts, CD4^+^/CD8^+^ ratio or in viral loads between IRIS-P and non-IRIS-P before treatment. This lack of difference in CD4^+^/CD8^+^ rate between the two groups may be due to the small sample size. The results indicated that all patients responded to ART, increasing CD4^+^ cell counts, which is in agreement with previous studies (20,21). It has been reported that the increase in CD4^+^ cell count with ART was not a risk factor for IRIS because it can occur without an appreciable CD4^+^ cell increase (22).

A previous study reported that a low CD4^+^/CD8^+^ ratio was an independent predictor for IRIS (23). They concluded that patients with a CD4^+^/CD8^+^ ratio less than 0.15 were more likely to have an IRIS event than were patients with a ratio greater than 0.30. However, in the present study CD4^+^/CD8^+^ ratios did not differ between IRIS-P and non-IRIS-P. In addition, 6 IRIS-P and 6 non-IRIS-P patients presented CD4^+^/CD8^+^ ratios less than 0.15 before ART.

The results showed that after one year of ART initiation, IL-6 plasma levels were significantly reduced in IRIS-P respect to pre-treatment values. Previous studies have also shown that ART decreases most markers of inflammation (24,25). The present study indicated that ART also caused a significant decrease in plasma levels of IL-18 in all the patients studied with respect to pretreatment values.

Before treatment, both IL-6 and IL-18 plasma concentrations in IRIS-P were significantly higher than in non-IRIS-P. Other authors also reported higher levels of inflammatory cytokines in IRIS-P than in non-IRIS-P previous to ART (24). The increased levels of these cytokines might be thought to be a characteristic of patients at risk of suffering from IRIS during ART. The higher values of IL-6 might reflect a resistance to glucocorticoids, which are normally involved in the decrease of the cytokine level and can promote a Th2 cytokine acquisition profile (15).

Interleukin-18 produced by macrophages is known to drive the differentiation of Th cells toward the Th1 type (6). It has been suggested that the higher levels of IL-18 in HIV-infected patients co-infected with TB may contribute to the sudden recovery of Th1 responses in those conditions (26). Thus, the higher IL-18 concentrations observed in IRIS-P before ART could also reflect this possibility. In the present study, an attempt to identify patients at potential risk of developing IRIS, a cut-off value of IL-18 ≥ 695 pg/mL before ART was chosen with 80% of specificity and 57% of sensitivity. Despite the fact that the study showed significant differences of IL-18 and IL-6 values between IRIS-P and non-IRIS-P, it would be necessary to carry out studies with larger number of patients to define cut-off values of both cytokines that could differentiate, with higher specificity and sensitivity, both types of patients from the general population of HIV patients prior to receiving ART, standardizing the pre-analytical and analytical variables.

Before ART, a positive correlation between CD4^+^ cell count and levels of cortisol was found in patients who did not suffer from IRIS. This correlation was suggested to indicate a more controlled clinical response of the HIV-infected patients (27). The results showed that plasma cortisol concentrations were similar before and after ART in all the patients studied. It was suggested that patients experiencing IRIS could present an inadequate HPA axis response (1). Previous reports have suggested an intra-adrenal shift from DHEAS towards the cortisol production during critical illness (28-30), as could be the case of HIV infected patients who suffered IRIS. It has been proposed that an exacerbated proinflammatory response could result from the suppression of the HPA axis and of adrenal failure or reflect glucocorticoid tissue resistance as well (31,32). This clinical disorder is known as critical illness-related corticosteroid insufficiency resulting from an inadequate corticosteroid production or action for such severe disease (32,33). In agreement with the previous idea, patients with adrenal insufficiency could present an altered regulation of the immune system, which has been linked to IRIS (34). The fact that plasma levels of DHEA-S, which are mainly of adrenal origin, did not significantly change in non-IRIS-P after ART, but rather decreased more than 20% in IRIS-P with respect to values before treatment, could reflect a more impaired adrenal function in IRIS-P than in non-IRIS-P. These results are consistent with other studies in chronic diseases, such as tuberculosis, suggesting that the decrease in DHEA-S levels were associated with worse prognosis of the disease (35). An inadequate adrenal steroid production could be thought to aggravate the inflammatory process, and to contribute to the development of IRIS in HIV-infected patients. Supporting this idea, the DHEA-S/Cortisol ratios were significantly lower in IRIS-P after ART than in the other patients. This decrease has been associated with the altered metabolic pathways of adrenocorticoids synthesis (33,34,36).

It has been reported that acquired immune deficiency syndrome patients with decreased levels of DHEA-S show excessive cytokine production by Th2 cells (IL-4, IL-5, IL-6, and IL-10) and suppression of other cytokines (IL-2, IFN-γ, IL-12) (37). This would negatively affect these patients’ evolution.

Abnormal thyroid function tests are more frequent in HIV-infected patients than in the general population (38). In the present study, the results showed that the plasma concentrations of T4 decreased significantly after ART, but the levels always remained within the normal range in all the patients.

Despite an intense search for hormonal or immune markers, which could predict which HIV-infected patients may be at risk of suffering IRIS after ART initiation, no reliable markers have been reported so far. The results of this study indicated that mean levels of IL-6 and IL-18 in IRIS-P almost duplicate the respective values in non-IRIS-P before ART. In addition, the decreased DHEA-S plasma levels and DHEA-S/cortisol ratio in IRIS-P with respect to values in non-IRIS-P, after ART initiation, could suggest a mild but critical adrenal deficiency in HIVinfected patients who undergo IRIS during ART. Based on these results, it could be useful to test the adrenal function of patients before they receive ART, in order to correlate the results with the potential development of IRIS.

In recent years, the enormous progress of ART has changed the survival expectations of HIV-infected patients. However, around 10% of the patients treated will suffer from IRIS during ART (18,39). Since the syndrome represents an important clinical problem, more studies on risk factors involving a larger number of patients, as well as the development of strategies for detection of patients at higher risk for IRIS are needed.
